# At what age should screening mammography be recommended for Asian women?

**DOI:** 10.1002/cam4.468

**Published:** 2015-04-27

**Authors:** Junko Tsuchida, Masayuki Nagahashi, Omar M Rashid, Kazuaki Takabe, Toshifumi Wakai

**Affiliations:** 1 Division of Digestive and General Surgery, Niigata University Graduate School of Medical and Dental Sciences1-757 Asahimachi-dori, Chuo-Ku, Niigata, 951-8510, Japan; 2 Department of Medical Oncology, Niigata University Graduate School of Medical and Dental Sciences1-757 Asahimachi-dori, Chuo-Ku, Niigata, 951-8510, Japan; 3 H. Lee Moffitt Cancer Center & Research Institute12902 Magnolia Drive, SRB 4.24012, Tampa, Florida, 33612; 4 Division of Surgical Oncology, Department of Surgery, Virginia Commonwealth University School of Medicine and the Massey Cancer Center1200 E. Broad Street, Richmond, Virginia, 23219

**Keywords:** Age, Asian women, breast cancer, ethnicity, screening mammography

## Abstract

Although regular screening mammography has been suggested to be associated with improvements in the relative survival of breast cancer in recent years, the appropriate age to start screening mammography remains controversial. In November 2009, the United States Preventive Service Task Force published updated guidelines for breast cancer, which no longer support routine screening mammography for women aged 40–49 years, but instead, defer the choice of screening in that age group to the patient and physician. The age to begin screening differs between guidelines, including those from the Task Force, the American Cancer Society and the World Health Organization. It remains unclear how this discrepancy impacts patient survival, especially among certain subpopulations. Although the biological characteristics of breast cancer and peak age of incidence differ among different ethnic populations, there have been few reports that evaluate the starting age for screening mammography based on ethnicity. Here, we discuss the benefits and harm of screening mammography in the fifth decade, and re-evaluate the starting age for screening mammography taking ethnicity into account, focusing on the Asian population. Breast cancer incidence peaked in the fifth decade in Asian women, which has been thought to be due to a combination of biological and environmental factors. Previous reports suggest that Asian women in their 40s may receive more benefit and less harm from screening mammography than the age-matched non-Asian US population. Therefore, starting screening mammography at age 40 may be beneficial for women of Asian ethnicity in well-resourced countries, such as Japanese women who reside in Japan.

## Introduction

**Figure 1:**
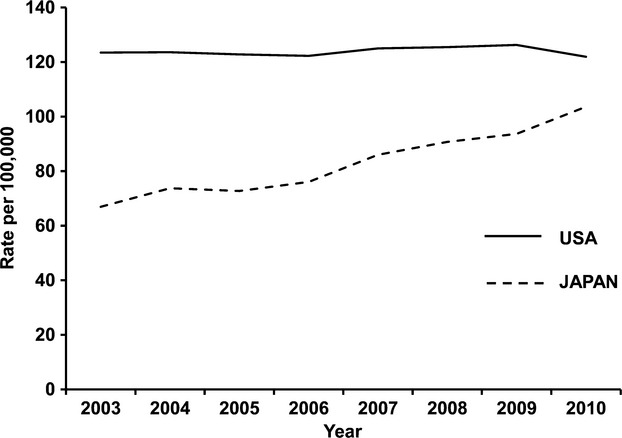
Trend in breast cancer incidence (2003–2010). Data for the US were obtained from age-adjusted SEER incidence rates by cancer site all ages all races female 2000–2011 (SEER) 2. Data for Japan were obtained from National Cancer Center Research Institute 17.

**Figure 2:**
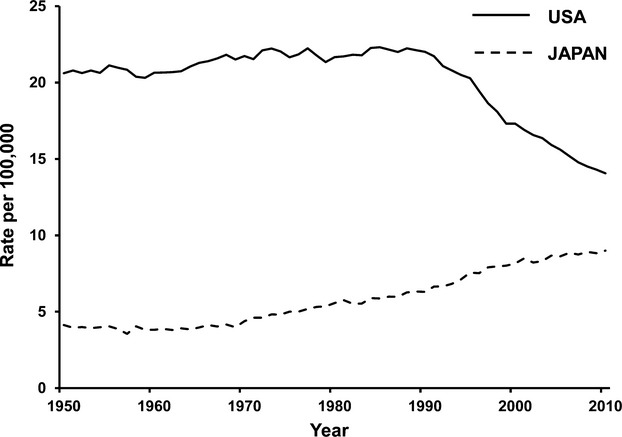
Breast cancer mortality age-standardized rate, all ages (1950–2011). Data were obtained from International Agency for Research on Cancer (IARC) 3.

Breast cancer is the most common cancer diagnosis, and the second most common cause of cancer death in US women 1. Although incidence remains high (Fig.1), mortality in the US has continued to decline since the early 1990s (Fig.2) 2–4. This improved relative survival for breast cancer has been attributed to the advances in systemic therapy as well as screening mammography. As a result of increased screening and earlier detection of breast cancer, the cases of early breast cancer have increased, which has also been associated with decreased mortality in the US 5.

Screening mammography has been shown to reduce breast cancer mortality by randomized controlled clinical trials (RCTs) 6–8. In fact, the breast cancer mortality reducing effect of screening mammography was reported by the United States Preventive Service Task Force (USPSTF) in 2002 9,10. Although the survival benefits of screening mammography have been established by RCTs, the appropriate patient age to start screening remains in question 11,12. In November 2009, the USPSTF published updated breast cancer screening guidelines that differed markedly from their last update in 2002 6,7,13. The new guidelines no longer support routine screening mammography for women ages 40–49 years. Instead, they defer the decision to screen in that age group to the choice of the patient and physician. [6,7,1, 13] The updated guidelines were different from others, including the American Cancer Society (ACS) guidelines; however, it remains unclear how the discrepancy affects patient survival, especially among certain subpopulations 13.

**Figure 3:**
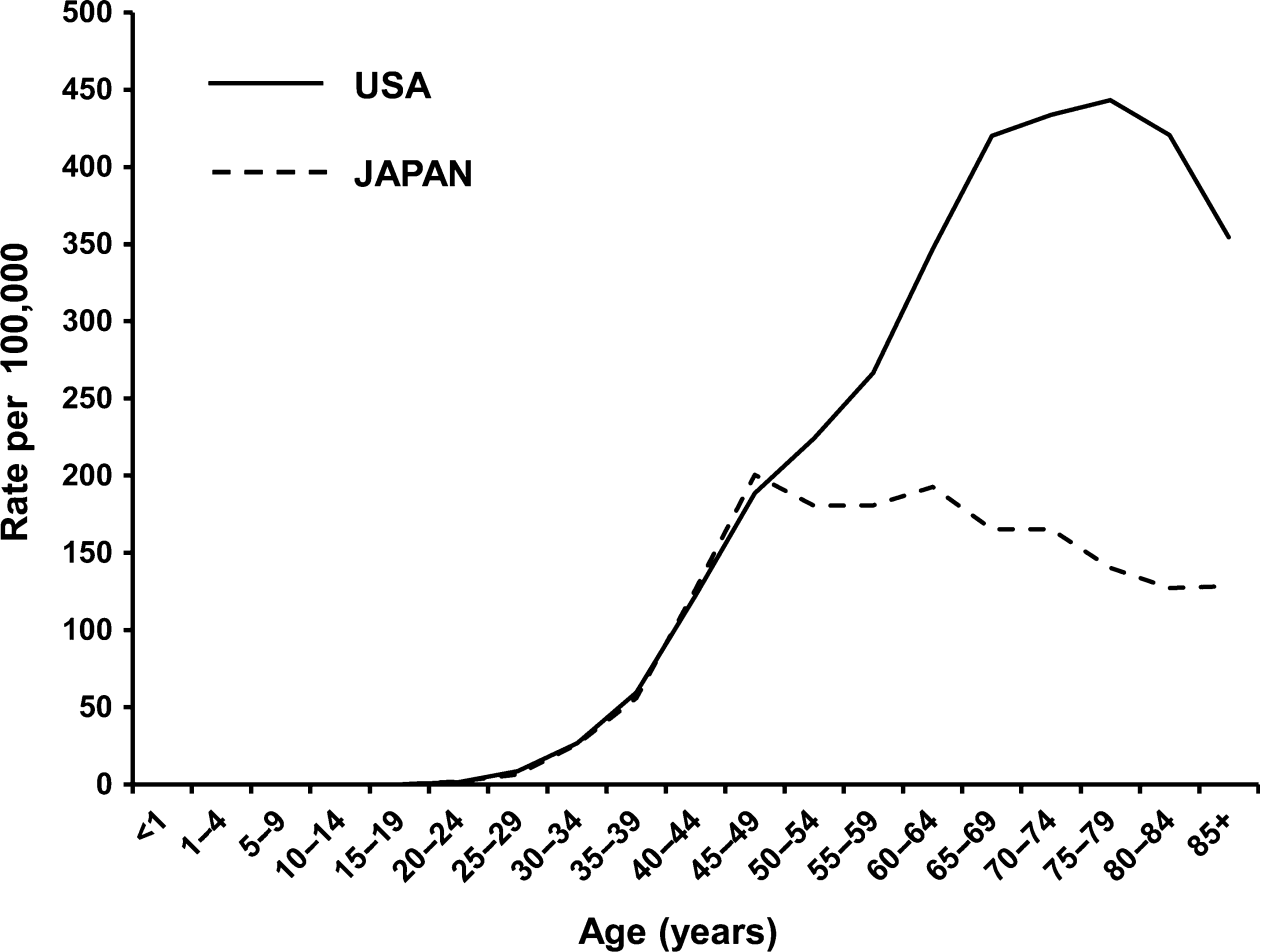
Breast cancer incidence by age group. Data for the US were obtained from age-Specific (Crude) SEER incidence rates by cancer site all ages all races female (2007–2011) 2. Data for Japan were obtained from National Cancer Center Research Institute (2010) 17.

Breast cancer incidence and mortality differ between women in the US and Japan (Figs.1 and 2). Interestingly, age-specific incidence curves also differ between the two populations (Fig.3). Although the biological characteristics of breast cancer differ by ethnicity, there have been few reports that discuss the starting age of screening mammography taking ethnicity into account 14,15. Here, we discuss the benefits and harm of screening mammography, and reconsider the starting age for screening mammography taking ethnicity into account, focusing on the Asian population.

## Incidence and Mortality of Breast Cancer Vary by Ethnicity; Should All American Women Be Screened the Same Way?

The incidence and mortality of breast cancer vary across countries and regions, with four to five-fold differences in incidence 16. Incidence and mortality of breast cancer are in general highest in North America and Europe, and lowest in Asia 14. However, as has been previously pointed out in the journal, Science, not only the incidence but also the mortality of breast cancer in Japan has been increasing since 1970 (Figs.1 and 2) 17,18. Today, breast cancer is the most common cancer diagnosed in Japanese women, and the fourth most common cause of cancer death among Japanese women 19. Increased exposure to risk factors, such as longer exposure to endogenous sex hormones, body mass index (BMI), and lower physical activity, is thought to affect the incidence of breast cancer in Japanese women 19. More importantly, however, it is consensus among Japanese researchers that one of the major reasons for the increasing mortality is a lower rate of screening mammography in Japan 20.

The overall screening mammography rate in the US across all ethnicities is 72.4% 21 and this high-examination rate has resulted in improved breast cancer survival 5. In contrast, the screening mammography rate in Japan is only 24.3% 22. Accordingly, it is difficult to evaluate the impact of screening mammography on breast cancer mortality when the rate of screening mammography is so low in Japan. Japanese researchers believe that the low rate of screening is partially due to a lack of appreciation for the importance of cancer screening within the general Japanese population 20,23. Given the differences in the screening mammography rates and the time-shift of breast cancer mortality between the US and Japan, and considering that similar advanced cancer therapies are available in both countries, it is likely that screening mammography plays an important role in explaining these differences in outcomes.

In addition to the differences in breast cancer incidence and mortality between women in the US and Japan, age-specific incidence curves also differ between the two populations (Fig.3). Older women have a higher incidence of breast cancer in the US, while the highest incidence was within the 45–49 age group in Japan 2,17. Because of this younger peak age in Japanese women, screening mammography in the 40–49 age group may provide a greater benefit in Japanese women.

## The Mortality Reduction Effect of Screening Mammography

To examine the effect of screening mammography on mortality, RCTs have been conducted in the US and Europe in the past several years 8,24–37. A meta-analysis of those RCTs evaluated the efficacy of screening mammography on the basis of the relative risk of breast cancer mortality 10. In 2002, the USPSTF reported that the relative risk of breast cancer mortality in the screening group during a 14-year observation period was 0.84 for all ages 7,8,10. In other words, breast cancer mortality was reduced by 16% in the screening group compared with the unscreened group 7,8,10. Because the US and many countries in Europe encourage screening mammography as a matter of national public health policy, the screening rate for breast cancer is high (70–80%) 8. These approaches have achieved a statistically significant decrease in breast cancer mortality due to the increased detection of early-stage disease 8.

Although the RCTs have established the survival benefits of screening mammography, breast cancer screening continues to be a topic of discussion 38–42. It has been suggested that the benefit of screening mammography has been overestimated by bias because the reports differ widely in the context and intensity of screening, as well as in the interpretation of the available evidence 39,43. Furthermore, some argue that the reduction in mortality might be due to advances in cancer therapies rather than screening mammography 39. However, such an argument does not account for the differences in the time-shift of breast cancer mortality between the US and Japan, where literally identical advanced cancer therapies are available in both countries. As a matter of fact, the 5-year relative survival rates of breast cancer patients by stage at diagnosis in Japan are as high as those in the US (Table1) 2,44. Nevertheless, breast cancer mortality in the US has continued to decline since the early 1990s, while the mortality in Japan has been increasing since 1970. One of the remarkable differences is the overall screening mammography rate in Japan is approximately one-third of that in the US, 24.3% versus 72.4%, respectively 21,22. Considering the differences in the time-shift of breast cancer mortality and the screening mammography rates between the US and Japan, screening mammography is thought to be one of the key factors that contributes to improved breast cancer mortality.

**Table 1:** Five-year relative survival by stage at diagnosis in the US and Japan.

	5-year relative survival (%)	
Stage at diagnosis	USA^1^	Japan^2^
Localized	98.5	98.2
Regional	84.6	84.5
Distant	25.0	28.2
All stages	89.2	89.1

1 Data were obtained from SEER 18 2004–2010 2.

2 Data were obtained from Monitoring of Cancer Incidence in Japan - Survival 2003–2005 44.

## Issues Regarding Starting Screening Mammography at age 40

The ACS recommends that average-risk women should begin annual screening with mammography at the age of 40 years 45. The ACS guidelines for breast cancer screening in average-risk women were last updated in 2003 46, and screening guidelines for women at very high risk were last updated in 2007 47,48. There is no specific upper age at which mammography screening should be discontinued 45. Rather, the decision to stop regular mammography screening should be individualized based on the potential benefits and harm of screening in the context of overall health status and estimated longevity 49. As long as a woman is in good health and would be a candidate for breast cancer treatment, she should continue to be screened with mammography 45. However, there is uncertainty as to the balance between the benefits and harm of screening mammography in women aged 40−49 years.

Interestingly, the USPSTF reports that the relative risk of death due to breast cancer for women randomly assigned to mammography screening was 0.78 for women aged 50 years or older, and 0.85 for those aged 40–49 years 7,8. This means that breast cancer mortality was reduced by 22% for women aged 50 years or older, and by 15% for women aged 40–49 7,8,10. The conclusions of the USPSTF in 2002 were as follows: “Based on fair evidence, screening mammography in women aged 40–70 years decreases breast cancer mortality 8. The benefit is higher for older women, in part because their breast cancer risk is higher.”^8^–^10^

The USPSTF reported their updated guidelines for screening mammography after a comprehensive assessment of the efficacy of breast cancer screening in terms of the net benefit, which is the sum of the benefits and harm of screening mammography 6,7. The benefits include a reduction in the risk of dying with breast cancer, less aggressive surgery and/or less aggressive adjuvant therapy and a greater range of treatment options when breast cancer is detected early. The harm of mammography includes radiation exposure, pain, anxiety, overdiagnosis, false-negative, and false-positive mammography results, and cost 6. Screening mammography for women aged 39–49 years had a 15% mortality reduction based on the results of eight meta-analysis studies 7,27,32,34–37. On the other hand, the harm, especially false-positive mammography, unnecessary additional imaging tests and histological examinations, were relatively greater in women aged 40–49 years when comparing the analyzed data with the data of the Breast Cancer Surveillance Consortium (BCSC) 7. Thus, the USPSTF recommended against routine screening mammography in women aged 40–49 years (grade C recommendation) 6. That recommendation, however, is not free from criticism 50, and the appropriate age for starting screening mammography remains controversial.

Most recently, the World Health Organization (WHO) produced a guideline named “WHO position paper on mammography screening” 43. This guideline recommended population-based screening mammography for women aged 40–49 years only if such a program is conducted in the context of rigorous research and monitoring and evaluation, if the conditions for implementing an organized program specified in this guideline are met, and if shared decision-making strategies are implemented so that women’s decisions are consistent with their values and preferences (conditional recommendation based on moderate quality evidence). On the other hand, WHO strongly recommends against the implementation of population-based screening programs for women aged 40−49 years in limited resource settings with weak or relatively strong health systems.

## Women Aged 40–49 in Japan May Have Less Harm from Screening Mammography than Age-matched Women in the US

In Japan, screening mammography, which was endorsed in 2000 for women aged 50 years and over, was expanded to include women aged 40–49 years in 2004 51. Breast cancer incidence increases in women after menopause in the US, while the highest incidence was seen in the 45–49 years age group in Japan (Fig.3). Therefore, it is thought that screening mammography in the 40–49 age group is important in Japan. However, at the time of that endorsement, data regarding the improvement in survival and the harm of screening mammography were not yet available 51. To address this issue, the harm of the screening mammography was investigated in Japanese women 51.

**Figure 4:**
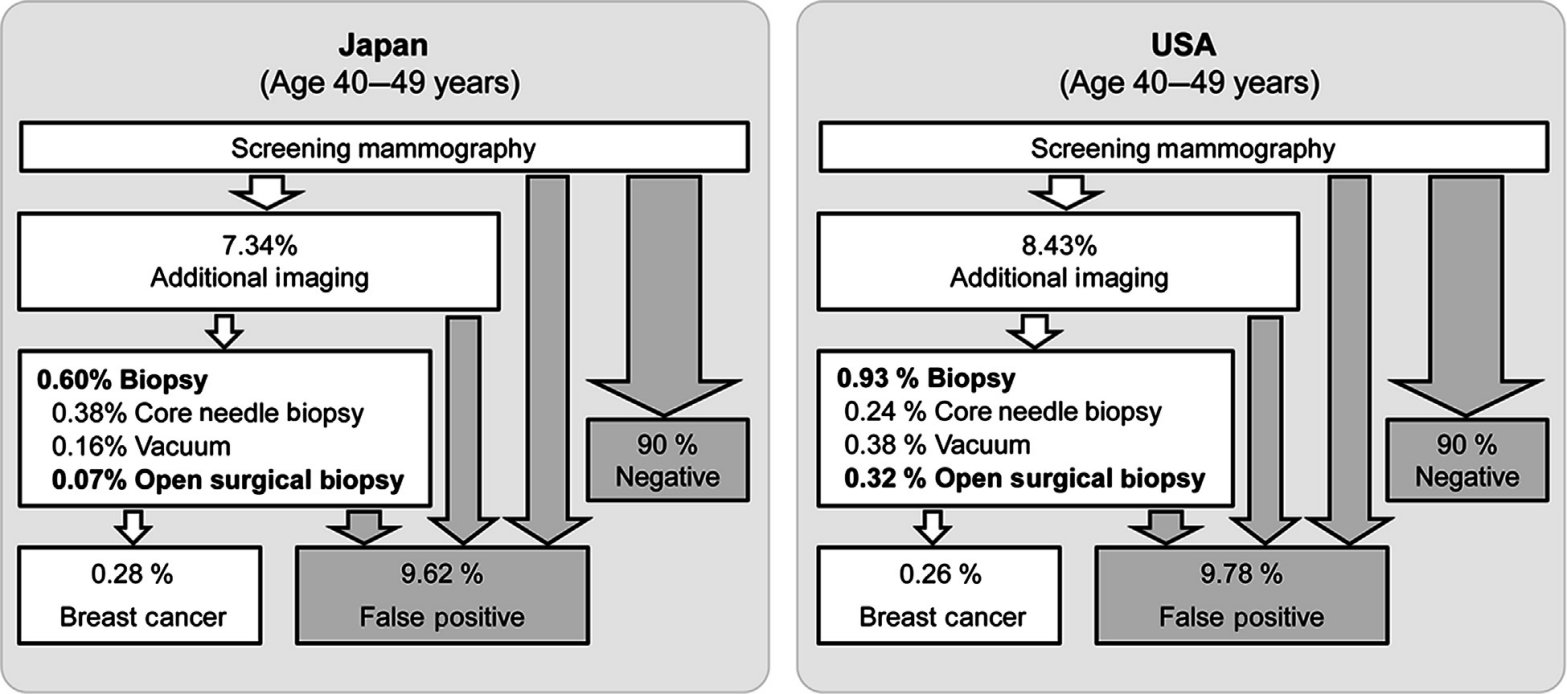
Comparison of the estimated number of additional imaging, FNA, biopsy and its procedures, false positives, and detected cancers per 1000 screened women in their 40s between Japan and the US. The data were reported by Kasahara et al. 51.

Kasahara et al.^51^ studied the harm of screening mammography using the initial test data collected from five prefectures in Japan. The analyzed harm included false-positive results, unnecessary additional imaging tests and biopsies, which were compared with US data 51. They collected screening mammography data from 144,848 participants from five Japanese prefectures to assess harm by age group (Fig.4) 51. The rate of cancer detection in the 40–49 age group was 0.28%. The false-positive rate (9.6%), rates of additional imaging by mammography (5.8%) and ultrasound (7.3%), fine needle aspiration cytology (FNA) (1.6%) and biopsy (0.6%) were higher in the 40–49 age group than in the other age groups 51.

The BCSC reported that the rate of cancer detection in the 40–49 age group was 0.26% in the US, which is similar to the data in Japan 51. The false-positive rate (9.8%) and rates of additional imaging (8.4%) were comparable to the data in Japan (9.6% and 7.3%, respectively) 51. The rate of biopsy was 0.9%, which appears to be higher than in Japanese reports (0.6%) 51. Interestingly, the open surgical biopsy rate in US was 0.32%, while the rate in Japan was only 0.07% 51.

What makes surgeons in the US decide to perform more open surgical biopsies? There are several possible reasons for this difference which may include medical and social factors. Adepoju et al.^52^ reported that factors associated with higher rates of surgical biopsy include younger age; Asian ethnicity; private insurance; small, rural, and non-teaching hospitals. Previous studies have shown that among women with breast cancer, younger women are more likely to present with a breast mass, and it is possible that these women would prefer to have complete excision of the mass. The reason for the higher rates associated with Asian ethnicity may be multifactorial: health literacy, socioeconomic status, patient preference, differential access to care and difference in imaging characteristics.

Interestingly, previous studies showed that reimbursement does impact what surgery is performed for breast cancer, since surgical biopsy has been shown to be more expensive than minimally invasive biopsies. Therefore, incentives by third-party insurance payers may be necessary to lower the rates of surgical biopsy. The increased rate of surgical biopsy was also associated with small, rural, and nonteaching hospitals. Other contributing factors include access to appropriate equipment and the limited technical expertise of regional surgeons and imaging subspecialists. Moreover, another factor includes “defensive medicine” which may incentivize a more aggressive surgical approach to biopsy out of fear due to the higher risk of being sued in the US than in Asia. Further studies examining these factors may be valuable to reveal the causes of higher surgical biopsy rates, and to increase the rate of minimally invasive biopsy.

The harm in terms of false positivity and the performance of unnecessary additional imaging did not show significant differences between the US and Japan 51. However, additional biopsies were much less frequent in Japan than in the US as reported by BCSC in all age groups 51. This may be explained by the difference in attitude towards aggressively pursuing additional open biopsies among surgeons between Japan and the US. Taken together, screening mammography appears to be less harmful in Japan than in the US. Considering that the highest incidence was seen in the 45–49 age group in Japan, Japanese women in their 40s appear to receive more benefit from screening mammography compared to the same age group in the US.

## Why Does Breast Cancer Incidence in Japanese Women Peak in the Fifth Decade?

**Figure 5:**
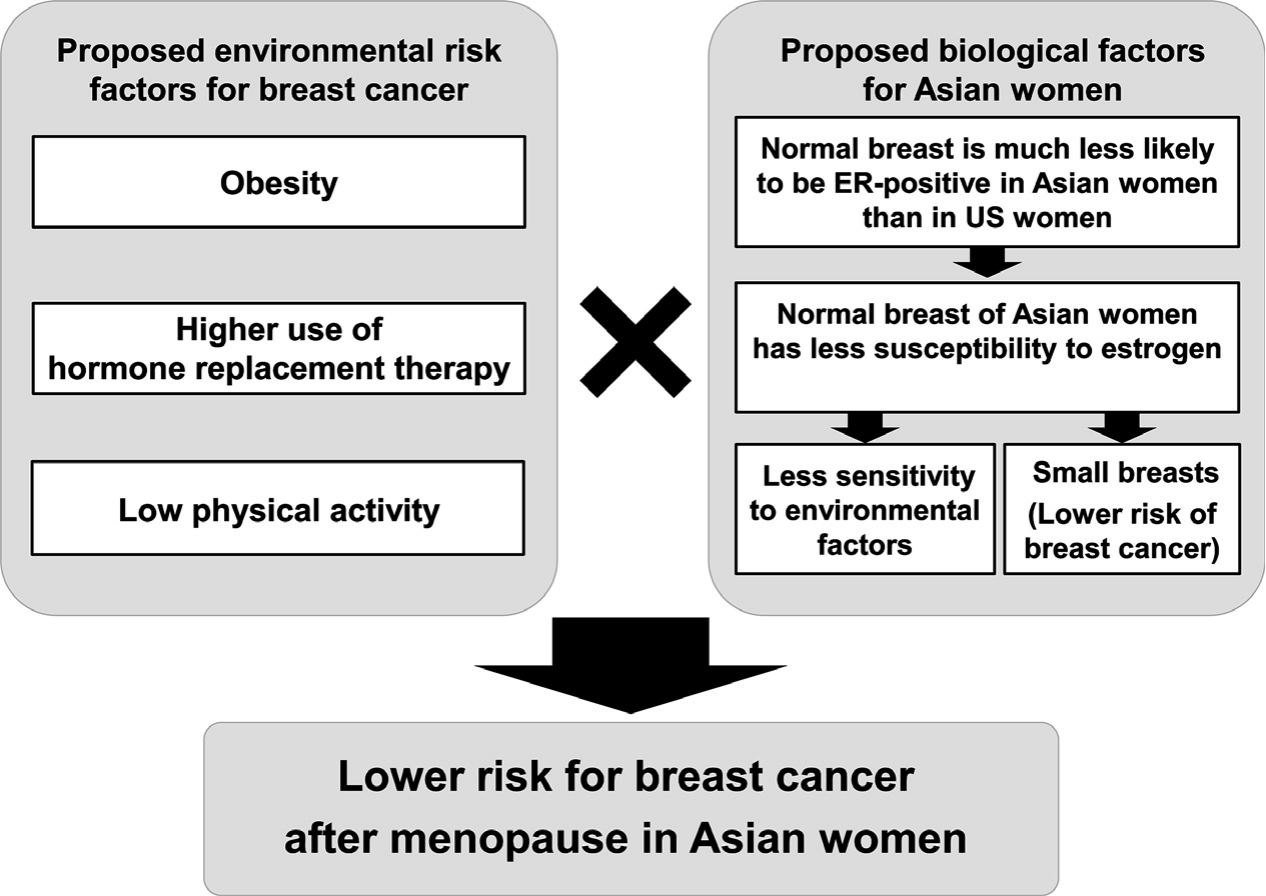
Proposed environmental risk factors for breast cancer and biological factors for Asian women. The reason why breast cancer incidence in Japanese women peaks in the fifth decade can be explained by a combination of the environmental and biological factors that may affect breast cancer incidence.

Breast cancer incidence increases in women after menopause in the US, while the highest incidence was seen in the 45–49 age group in Japan 4. The difference in peak age of breast cancer incidence in Japan can be explained by a combination of environmental and biological factors that may affect breast cancer incidence (Fig.5) 14. There are significant differences in the distribution of risk factors for postmenopausal breast cancer, particularly the high prevalence of obesity and use of hormone replacement therapies in the US compared to Japan (Fig.5) 4,53,54. It has been reported that normal breast tissue is much less likely to be ER-positive in Japanese women than in US women 14,55. Therefore, it is suggested that normal breast tissue of Japanese women has less susceptibility to estrogen 14. Indeed, there is a significantly lower prevalence of postmenopausal estrogen receptor (ER)-positive breast cancer in Japanese women living in Japan compared with Western populations 14. ER-positive breast cancer is sensitive to epidemiological risk factors, including parity, age at first partum, and BMI, while ER-negative cancer is somewhat less sensitive 14. Taken together, the combination of environmental and biological factors appears to impact the differences in age of peak breast cancer incidence between Japanese and US women.

## Peak Age of Breast Cancer Incidence in Asian American Women Is Similar to Japanese Women; Asians Comprise About 5% of the Overall US Population

As described above, the highest incidence of breast cancer occurred in the 45–49 age group in Japan, probably due to a combination of biological and environmental factors. What about other Asian populations? Interestingly, many studies in other Asian countries also observed that age-specific incidence rates peaked at around age 50 and then declined with age. This trend was observed not only in Japan, but also in Korea, Taiwan, Hong Kong, and Singapore 14. It has been reported that ER positivity among breast cancer patients in Asia was also lower than in Western women 56,57. These findings are important to consider in the West as well, as there are Asian populations also in the US. According to population demographic statistics, Asian Americans comprise about 5% of the overall US population 58. For those women of Asian ethnicity in the US, we believe that starting screening mammography in the fifth decade may provide a similar benefit as it has in Japan.

## Conclusions

The peak age of breast cancer incidence differs between US and Asian women. Currently, both environmental and biological factors are proposed to be the causes for this difference. Asian women in their 40s appear to receive more benefit from screening mammography compared to age-matched non-Asian women in the US. Therefore, we recommend that screening mammography start at age 40 for women of Asian ethnicity, such as Japanese women who reside in Japan. Since the effectiveness of routine screening relies on societal resources, which is the basis of WHO and USPSTF recommendations against routine screening in the age 40–49 group, our recommendation may therefore apply to well-resourced countries. For example, our results implicate an intriguing possibility that our recommendation may be applicable for ethnically Asian women in the US. Although the benefit of early screening for these women needs to be proven by a large prospective study, our results suggest that ethnicity and environment should be taken into account when considering screening mammography as a public health measure.

## Conflict of Interest

None decalred.
